# Lactobacillus Supplementation Modulates Rumen Microbiota and Metabolism in Yaks Under Fattening Feeding Conditions: A Comprehensive Multi-Omics Analysis

**DOI:** 10.3390/ani15121681

**Published:** 2025-06-06

**Authors:** Jianlei Jia, Pengjia Bao, Ning Li, Siyuan Kong, Min Chu, Qian Chen, Ping Yan

**Affiliations:** 1 Key Laboratory of Yak Breeding Engineering Gansu Province, Institute of Husbandry and Pharmaceutical Sciences, Chinese Academy of Agricultural Sciences, Lanzhou 730050, China; jiajianlei87@163.com (J.J.); baopengjia@caas.cn (P.B.);; 2 Institute of Western Agriculture, Chinese Academy of Agricultural Sciences, Changji 831100, China; lining06@caas.cn; 3 School of Life Sciences, Qilu Normal University, Jinan 250200, China; chenqian87@163.com; 4 Agricultural Genomics Institute at Shenzhen, Chinese Academy of Agricultural Sciences, Shenzhen 518100, China; kongsiyuan@caas.cn

**Keywords:** Lactobacillus supplementation, rumen microbiota, multi-omics analysis, yak metabolism, high-energy diet

## Abstract

The rumen is a critical organ that facilitates nutrient digestion in ruminant animals. However, the biological mechanisms by which rumen microbiota and its metabolites enable Lactobacillus to modulate rumen structure and maintain functional homeostasis under fattening feeding conditions remain poorly understood. In this study, 80 male Pamir yaks were selected, and a 170-day data collection phase was implemented. Correlation phenotypic data and multi-omics analyses (rumen microbial sequencing and rumen epithelial metabolomics) were conducted to investigate the regulatory effects of Lactobacillus supplementation on rumen microbiota and metabolic processes in a concentrate-based rearing yak model. The results demonstrated that feeding a high-energy diet may impair yak ruminal histomorphology, microbiota composition, and function while negatively modulating rumen microbiota–metabolic profiles associated with specific ruminal microbial communities and functions. Lactobacillus intervention treatment optimized the yak ruminal microbiome composition (mucous layer maturation was promoted, *Prevotella* and *Ruminococcus* abundance were reduced, and *Fibrobacter* and *Muribaculaceae* abundance were increased), thereby altering metabolite concentrations involved in various metabolic pathways under a high-energy feeding pattern (fatty acid metabolism pathways were upregulated). These alterations elucidated the beneficial impacts of the Lactobacillus supplementation strategy on yak ruminal health without compromising the high-energy intensive rearing pattern. Furthermore, the regulated ruminal microbiome metabolites may serve as potential biomarkers for future investigations into the functional impacts of Lactobacillus intervention treatment on healthy feeding strategies for yaks.

## Simple Summary

The rumen is a critical organ that facilitates nutrient digestion in ruminant animals. In this study, male Pamir yaks were employed to establish a concentrate-based rearing model with Lactobacillus intervention. A systematic exploration was carried out encompassing rumen tissue histomorphology, fermentation parameters, 16S rRNA sequencing of the microbiome, and LC-MS non-targeted metabolomics to delve into the regulatory effects of Lactobacillus supplementation on rumen microbiota and metabolic processes in yaks under fattening conditions. The targets of Lactobacillus within rumen microbiota metabolism were identified, and the correlations and interactions between the ruminal microflora and differential metabolites were analyzed. This research provides a more theoretical guidance for yak fattening during the hay period and contributes to enhancing economic benefits for herdsmen.

## 1. Introduction

Yaks (*Bos grunniens*), as a unique domestic species in the plateau regions, possess a special gastrointestinal environment to adapt to the hypoxia and long periods of grass withering in the plateau grazing conditions [1]. The forage and nutrients are inadequate to satisfy the maintenance requirements of the livestock in the cold seasonal stage, especially in livestock reared under traditional grazing approaches [2]. Therefore, a feeding strategy primarily focused on supplementary concentrate has progressively supplanted the traditional grazing-based rearing system to enhance production efficiency. A study has proven that ruminants necessitate an ingestion of 40% to 70% dietary roughage for the upkeep of normal gastrointestinal functionality and the homeostasis of microbiota [3]. Nevertheless, some livestock farmers, in pursuit of higher economic benefits brought by greater daily weight gain, have raised the dietary concentration for yaks to more than 75% [4,5]. Persistent utilization of this feeding model will likely have a profound and detrimental impact on the well-being of yaks, possibly resulting in the metabolic dysfunction of rumen microorganisms, a rapid accumulation of volatile fatty acids (VFAs), and a decline in pH values [6]. The transfer of fermentable carbohydrates into the small intestine through the rumen can trigger intestinal acidosis, modify the gut microbial community structure, and undermine the integrity of intestinal epithelial morphology [7]. Consequently, the development of a sustainable feeding system for domestic yaks that maintains an equilibrium between efficient energy intake and gastrointestinal health represents a critical strategy for promoting their healthy cultivation.

The morphological and functional integrity of the rumen serves as a crucial determinant for ruminant production performance and health status. The management of rumen health is regulated by a plethora of factors, encompassing feeding conditions, dietary composition, and microbial communities [8]. The ruminant rumen constitutes a vast and dynamic ecosystem teeming with diverse microorganisms [9]. This ecosystem undergoes evolution in response to animal growth and development, as well as variations in the type or nutrient composition of daily diets [10]. Rumen microbiota play a vital role in decomposing complex food components such as dietary fiber and also regulate the metabolism of bile acids, lipids, and amino acids through metabolite interchange with the host [11]. The high-energy diet (HED) and high-fat diet (HFD) are common strategies for short-term rapid fattening, but they are typically accompanied by the remodeling of the gastrointestinal microbiota. Researchers have demonstrated that an increase in the dietary concentrate is negatively correlated with the relative abundance of dominant ruminal microbial communities. HED and HFD, as dietary interventions, modify rumen microbiota and reprogram metabolism, encoding a differential short-chain fatty acids (SCFAs) profile, which leads to alterations in the host metabolism, such as the upregulation of SCFAs transporter genes expressed by the ruminal epithelium to significantly deplete the ratio of acetic acid to propionic acid [12,13]. SCFAs have been recognized as significant mediators in maintaining rumen homeostasis by participating in signaling pathways that modulate host gene expression and uphold rumen barrier homeostasis [14]. Additionally, previous studies have ascertained that over 60% of the total metabolites produced during rumen fermentation can be attributed to microbial activity, and rumen microbiota confer host resistance to HFD-induced weight gain by modulating dietary polyunsaturated fatty acid (PUFA) metabolism [15,16]. Nevertheless, there remains a scarcity of valuable insights addressing the adverse effects associated with HED or HFD on hosts, ruminal microbiota dynamics, and metabolic functions, particularly in yaks.

In early research, it has been demonstrated that the probiotic supplement can effectively facilitate maintaining a stable and salutary gastrointestinal microbiota in animals [17]. A multiplicity of probiotics, especially Clostridium and Bacillus species, generate short-chain fatty acids (SCFAs), similar to experiencing a phenomenon dubbed “colonization resistance” in the rumen, which are the major metabolic products of gastrointestinal microbiota derived from dietary fiber [18]. For several decades, Lactobacillus has been among the most profoundly investigated beneficial microorganisms, given its fundamental role in diverse biological processes and ecosystems, particularly with regards to fermented feed and animal health cultivation [19]. Recent studies have indicated that Lactobacillus ranks among the most prominent beneficial bacteria present in the ruminants’ rumen, offering a wide range of health benefits [20]. These benefits encompass the regulation of the gastrointestinal microbiota, peristalsis stimulation, preservation of the micro-ecological equilibrium, enhancement of the overall functionality, improvement in fodder utilization, and the promotion of animal health production [21]. Moreover, Lactobacillus species are non-toxic, biodegradable, and biocompatible, presenting themselves as suitable alternatives to conventional probiotic preparations [22]. Lactobacillus species are regarded as components of the transient gastrointestinal microbial assemblage, originating from the external environment, with dietary intake serving as the predominant source, and they interact on a daily basis with the more persistent members of the gastrointestinal microbiome [23]. Research studies have found that a profusion of metabolites produced by Lactobacillus are directly implicated in the host metabolic system; these act as detoxifying agents by mediating the saturation of PUFAs derived from dietary fats through the production of antimicrobial peptides and SCFAs, which augment the residential time of probiotics in the rumen and curtail aerobic bacteria within the gastrointestinal microbiota to escalate ruminal fermentation efficacy [24]. However, yaks (*Bos grunniens*), a keystone species endemic to the Tibetan Plateau, exhibit unique gastrointestinal adaptations to hypoxia, low temperatures, and scarce forage [25]. Their gut microbiota is intrinsically linked to these survival strategies, yet probiotic interventions tailored to their distinct microbial ecosystems remain underexplored.

To better understand the interactions between Lactobacillus and the “microbe–host metabolism” to optimize the rumen microecosystem, male Pamir yaks were employed to establish a concentrate-based rearing model with Lactobacillus intervention. A systematic exploration was carried out, encompassing rumen tissue histomorphology; fermentation parameters; microbiome and metabolomics to delve into the regulatory effects of Lactobacillus, which secrete organic acids and antibacterial active substances within the rumen environment; the crucial role of optimizing the internal conditions of the rumen as well as the ratio of VFAs; and the regulation of the colonization of microorganisms involved in cellulose degradation and nutrient utilization, to mediate the host regulation of metabolic pathways such as those related to SCFAs or/and PUFAs via metabolic products. It was demonstrated that the molecular mechanisms by which Lactobacillus exerts its activity was associated with high-energy dietary effects involve the modulation of rumen microbiota function and the preservation of rumen barrier integrity. This research provides a more theoretical guidance for yak fattening during the hay period and contributes to enhancing economic benefits for herdsmen.

## 2. Materials and Methods

All procedures involving the use of animals were approved by the Animal Care Committee at the Lanzhou Institute of Animal Science & Veterinary Pharmaceutics, the Chinese Academy of Agricultural Sciences, China (20230813-031). Furthermore, approval for the slaughtering of the animals was obtained in accordance with the National Administration of Experimental Animal Slaughtering and Quarantine Regulations.

### 2.1. Animal Models and Experimental Design

The Junmahong Livestock Farm, situated in the Tajik Autonomous County of Taxkorgan, Xinjiang, China, served as the experimental venue for this research. Eighty male Pamir yaks, aged within the range of 4 to 5 years and weighing between 250 and 280 kg, were enlisted as study subjects. These yaks foraged on the natural pastures of the Pamir Plateau. All yaks in the experimental group were accommodated in a roofed shed under intensified rearing circumstances, while those in the control group continued their grazing during the experimentation. This study was conducted for a period of 170 days, commencing on 1 November 2023 and concluding on 18 April 2024. The initial 20 days constituted an adjustment phase for the experimental group, during which the proportion of the concentrated feed was incrementally escalated. The subsequent 150 days were dedicated to the data collection phase. The feeding strategies were formulated based on the determination of the feed intake of the grazing Pamir yaks and were in accordance with the recommendations stipulated in the Chinese Beef Cattle Raising Standard (NY/T 815-2004) [26]. In alignment with the experimental design, the requisite concentrated feeds for the experiment were custom-manufactured by Teamgene (Shandong) Agricultural Technology Co., Ltd. (Zibo, China). The experimental dietary regimen was a well-mixed ration consisting of concentrated feed and roughage feed (wheat grass) in an equal ratio. The composition of the ingredients and nutritional levels are presented in Table 1. The nutrient composition of the mixed diet was ascertained by the laboratory at Qilu Normal University by employing ‘Feed Analysis and Quality Test Technology’ (Yang, 1993 [27]).

Subsequently, using the SRS method of the SAS software (Version 9.4), the yaks were randomly divided into four groups based on body weight (20 in each group). All experimental yaks were subjected to one of the following dietary treatments: a low-energy diet group (LEG: NEG 2.12 MJ/kg, with a daily intake of 5 kg of the mixed diet per capita; the experimental control group), a high-energy diet group (HEG: NEG 2.69 MJ/kg, with 5 kg of the mixed diet per capita per day), and a high-energy diet with Lactobacillus supplementation group (HLG: NEG 2.69 MJ/kg, with 5 kg of the mixed diet per capita per day and administered 0.02% of the Lactobacillus (0.2 g/kg feed) powder throughout the entire experimental period). The control group’s yaks grazed on the pasture during the experimental period (CKG: NEG 0.11 MJ/kg, with a daily intake of 5 kg of hay per capita; the blank control group). The Lactobacillus was purchased as a commercial strain (Lactobacillus fermentum) from Wei’er Bioengineering Co., Ltd. (Tai’an, China).

### 2.2. Sample Collection and Measurement

In the present study, yak feeding was conducted at 10 a.m. and 6 p.m. (Beijing Time), and all yaks were scheduled for slaughter at 8 a.m. (after the 10 a.m. feeding session of the previous day, that is, they were subjected to fasting until slaughter following weighing). All yaks were subjected to weighing under a fasted condition on the 1st and 170th days of the experimental period. The initial body weight (IBW) and final body weight (FBW) were accordingly registered. The average daily growth (ADG) formula was ‘ADG (g/d) = (FBW − IBW)/trial days’.

From each group, the experimental yaks were narcotized using a captive bolt pistol and subsequently exsanguinated at the end of the experiment. Immediately following slaughter, the rumen was isolated immediately. Rumen contents were retrieved from the dorsal sac, filtrated through four layers of sterile cheesecloth, and then centrifuged at 350× *g* for 15 min at 4 °C. The obtained rumen fluid was apportioned into three 15 mL centrifuge tubes and stored at −80 °C for subsequent sequencing analysis and volatile fatty acid (VFA) determination. Once thawed to ambient temperature, the rumen fluid was centrifuged again at a rotational speed of 12,000× *g* for 15 min at 4 °C. Subsequently, 1 mL of the supernatant was transferred into a 1.5 mL centrifuge tube, followed by the addition of 0.2 mL of a 25% metaphosphoric acid solution incorporating the internal standard (2-ethylbutyric acid, 2EB). Another round of centrifugation was carried out under the same conditions (12,000× *g* for 15 min at 4 °C). The concentration of volatile fatty acids (VFAs) was measured by gas chromatography (GC-2014, Shimadzu Corporation, Kyoto, Japan), in accordance with well-recognized methodologies.

Four specimens were respectively harvested from the dorsal and ventral domains of the rumen. The segregated rumen tissue was immobilized in 4% buffered formaldehyde for a period of 72 h. Subsequently, the specimens underwent dehydration via diverse concentrations of glucose and were subsequently embedded in a cryogenic embedding medium. Tissue sections with a thickness ranging from 5 to 7 µm were fabricated and stained with hematoxylin and eosin (HE), facilitating the discernment of villous height, villous width, crypt depth, mucosal thickness, and muscular thickness. Images were obtained using the DP70 software (version 03.03) (Olympus, Nagano, Japan) for each transverse section of the rumen tissue. The slides were inspected using a BX51 microscope (Olympus, Nagano, Japan). The measurements were obtained using Image Proplus 5.1 software; ten well-oriented and intact crypt-villus units in each slide were measured in triplicate.

Total DNA extraction from the microbial consortia present in the rumen fluid samples, in conjunction with library establishment and microbiome sequencing, was executed by OE Biotech Co., Ltd. (Shanghai, China). Each group encompassed six replicates. For the evaluation of microbial diversity and community composition, sequencing of the 16S rRNA gene was implemented using the Illumina NovaSeq/Hiseq Xten platform (Illumina Inc., San Diego, CA, USA). PCR amplification targeting the V3–V4 hypervariable region of the microbial 16S rRNA gene employed forward primers (5′-CCTACGGGNGGCWGCAG) and reverse primers (5′-GGACTACHVGGGTATCTAAT). The thermocycling conditions comprised an initial denaturation stage at 95 °C for 2 min, succeeded by 35 cycles encompassing denaturation at 95 °C for 2 min, annealing at 72 °C for 30 s, and elongation at 72 °C for 5 min.

Metabolomics analysis was performed by OE Biotech Co., Ltd. (Shanghai, China), with each group encompassing twelve replicates. The metabolic profiles of the rumen fluid samples were characterized using an Agilent 1290 Infinity LC system (Agilent Technologies, Santa Clara, CA, USA) coupled with an AB SCIEX Triple TOF 6600 System (AB SCIEX; Framingham, MA; USA). An ACQUITY UPLC BEH Amide column with a particle size of 1.7 µm and dimensions of 2.1 × 100 mm was utilized for the chromatographic separation in both the positive and negative ionization modes. The mobile phase A constituted a mixture of 25 mM ammonium acetate and ammonium hydroxide in water, while mobile phase B consisted of acetonitrile.

### 2.3. Statistical Analysis

The analysis of 16S rRNA was accomplished by using R software (version 3.1.2) in conjunction with QIIME software (version 1.9.1) and UPARSE software (version 11). Adhering to the UPARSE pipeline, multiplexed reads were amalgamated into operational taxonomic units (OTUs) based on a 97% sequence similarity threshold. The classification of the 16S rRNA gene sequences was implemented using the RDP Classifier (version 2.2). The OTUs were appraised via various metrics for alpha diversity analysis, encompassing OTU rank curves, rarefaction analyses, and indices such as Shannon, Chao1, Simpson, and ACE. For beta diversity evaluation, principal coordinates analysis (PCoA) and the unweighted pair group method with arithmetic mean (UPGMA) were executed by employing weighted UniFrac distances within the QIIME framework. Subsequently, PICRUSt was harnessed to predict microbial functionality. The bacterial domains, phyla, and genera were compared through the Wilcoxon rank-sum test; a false discovery rate (FDR)-adjusted *p* value less than 0.05 was regarded as statistically significant.

Raw data from UPLC-Q-TOF/MS were converted to mzXML files by the means of the Proteo Wizard MSconvert tool and subsequently processed using XCMS online software (Version 4.6.0; https://bioconductor.org/packages/release/bioc/html/xcms.html, accessed on 1 May 2024). The parameters for XCMS encompassed the following settings: feature detection centwave settings (Δm/z = 25 ppm; peakwidth = c(10,60)); retention time correction obiwarp settings (profStep = 1); and minfrac parameters set at 0.5 along with bw = 5 and mzwid = 0.025 for chromatogram alignment. Following normalization and integration via support vector regression methodologies, the processed data were uploaded into MetaboAnalyst version 4.0 for further assessment (www.metaboanalyst.ca). Orthogonal partial least squares discriminant analysis (OPLS-DA) in conjunction with three-dimensional principal component analysis (3D-PCA) for both positive and negative models was conducted subsequently to log the transformation and Pareto scaling operations. For each variable within the OPLS-DA models, variable importance projection values (VIPs) were computed to determine their contribution to classification efficacy; metabolites presenting VIP values greater than one underwent further scrutiny via a Student’s *t*-test at a univariate level where *p* < 0.05 signified statistical significance.

Prior to undertaking any statistical appraisals, all datasets underwent meticulous outlier inspection either by delineation or assessment using box-and-whisker plots in conjunction with Shapiro–Wilk tests to validate normal distribution assumptions. The results are presented as means ± SME across the groups analyzed under a completely randomized experimental design by utilizing the Statistical Analysis System software version 9.4 (SAS Inc.; Raleigh, NC, USA). To identify significant differences among groups, the LSR-SSR method was applied for normal distribution data, otherwise by the Kruskal–Wallis method. A significance level of *p* < 0.05 was established, with *p* < 0.1 indicating a trend.

## 3. Results

### 3.1. Changes in Yak Weight Is Linked to the Modulation of HED-Induced Changes by Lactobacillus in the Rumen Structure and Functional Homeostasis

**Figure 1:**
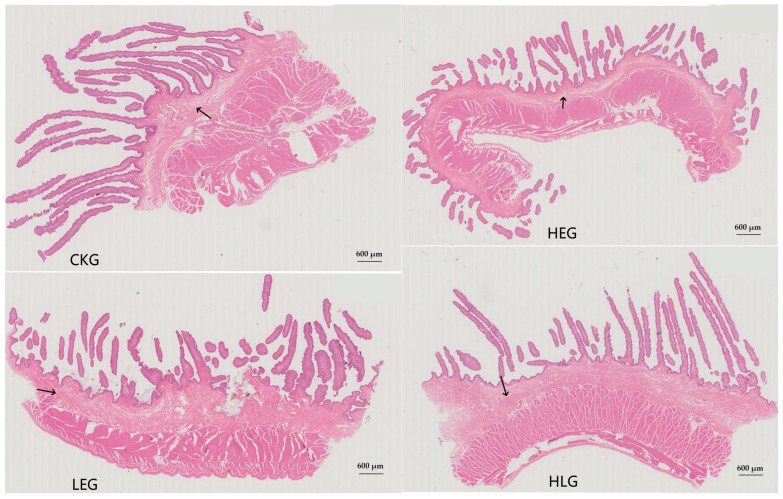
Rumen tissue morphology for the yak diets of CKG (0.11 MJ/kg NEG), LEG (2.12 MJ/kg NEG), HEG (2.69 MJ/kg NEG), and HLG (2.69 MJ/kg NEG + 0.02% Lactobacillus). The arrow was the maturity of the mucosal layer. 600 µm.

Based on the experimental design requirements, four distinct treatment groups of the yaks were established. As anticipated, the growth performance indicators of the yaks (Table 2), including final body weight (FBW), total gain (TG), and average daily gain (ADG), exhibited a significant increasing trend with a rise in dietary energy levels (*p* < 0.05). Furthermore, these indicators differed significantly among the groups subjected to Lactobacillus and energy treatments. Notably, both FBW and ADG were significantly higher in HLG compared to HEG (*p* < 0.05). With the addition of Lactobacillus, the TVFA, acetate, acetate/propionate and MCP concentrations increased significantly (*p* < 0.05). The concentrations of propionate, isobutyrate, butyrate, and pentanoate were higher in HLG than in HEG, although the differences failed to reach statistically significant levels (Table 3). Additionally, the rumen epithelium developed significantly due to Lactobacillus supplementation. The stratum corneum, tunica propria, mucosal epithelial thickness, submucosal thickness, muscle layer thickness, epithelial papillae density, epithelial papillae length, and epithelial papillae width increased significantly in HLG compared with HEG, and the degree of development was more similar to that of that in LEG and CKG (Table 4 and Figure 1). These findings suggest that the addition of Lactobacillus may contribute to the production of substances, such as organic acids and bacteriocins in the rumen, which could improve the rumen microbial community structure, promote the recovery of mucous layer maturation, enhance rumen digestion efficiency, and facilitate nutrient digestion and absorption, thereby effectively optimizing growth performance indicators. Notably, the rumen structure and functional homeostasis in the Lactobacillus concurrent feeding group, in the presence of HED, were observed to be in alignment with normal feeding patterns.

### 3.2. HED and Lactobacillus Intervention Are Linked to Compositional Rumen Microbiota Changes

**Figure 2:**
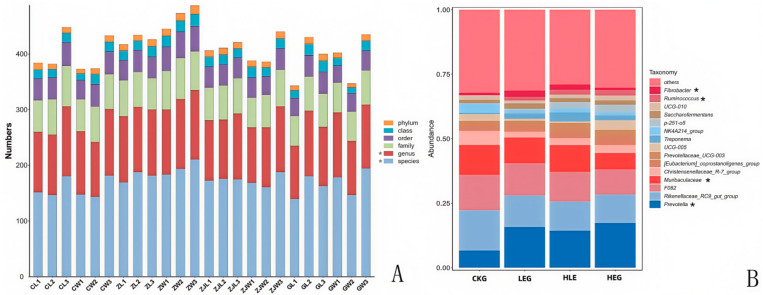
Classification of the bacterial community composition for the yak diets of CKG (0.11 MJ/kg NEG), LEG (2.12 MJ/kg NEG), HEG (2.69 MJ/kg NEG), and HLG (2.69 MJ/kg NEG + 0.02% Lactobacillus). (**A**) Classification of the microorganisms’ levels. (**B**) The relative abundance of the top 10 genera of the ruminal microbiome composition profiles revealed by 16S rRNA sequencing (each color represents one bacterial genus). * Significant correlation among the differential treatments (*p* < 0.05).

**Figure 3:**
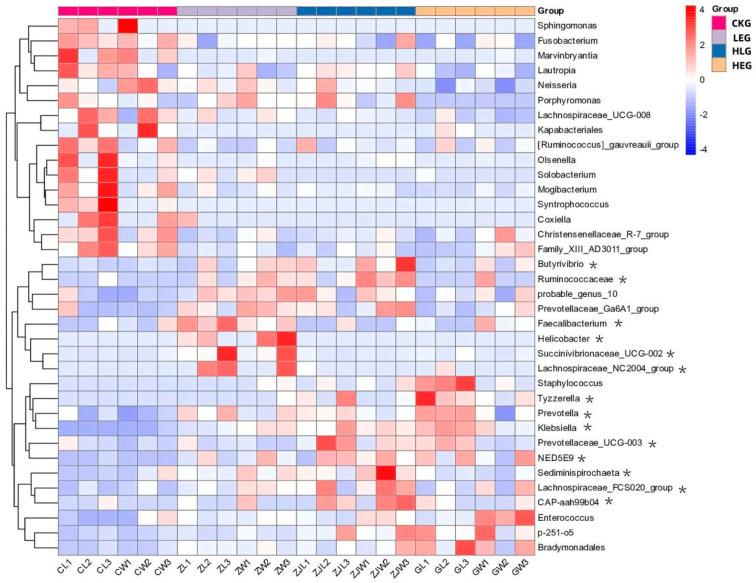
Predicted microbial composition using a heatmap for the yak diets of CKG (0.11 MJ/kg NEG), LEG (2.12 MJ/kg NEG), HEG (2.69 MJ/kg NEG), and HLG (2.69 MJ/kg NEG + 0.02% Lactobacillus). * Significant correlation among the differential treatments (*p* < 0.05).

**Figure 4:**
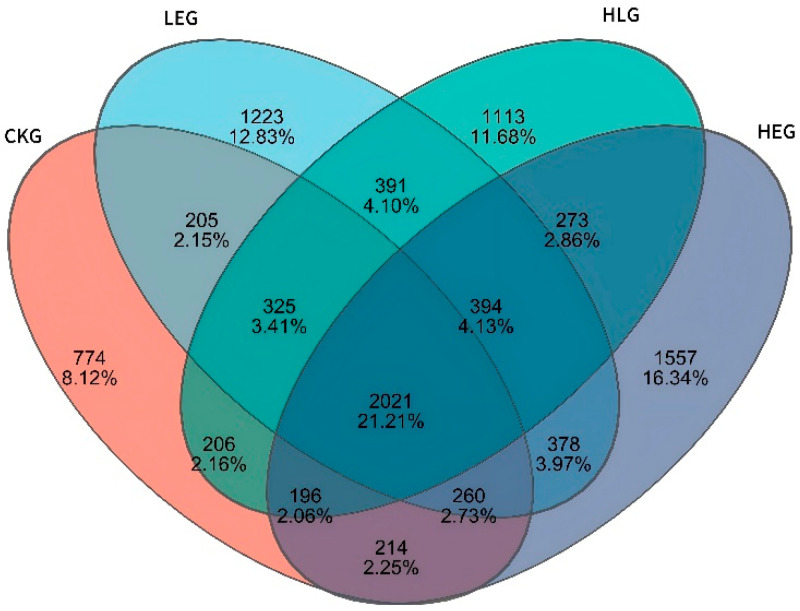
Ruminal microbiota Venn diagram for the yak diets of CKG (0.11 MJ/kg NEG), LEG (2.12 MJ/kg NEG), HEG (2.69 MJ/kg NEG), and HLG (2.69 MJ/kg NEG + 0.02% Lactobacillus).

Given that the rumen microbiota, rumen fermentation parameters, and epithelial morphology are known to modify the composition of the microbiota during HED-mediated processes, we investigated the evolution of rumen microbiota composition under the influence of Lactobacillus throughout this process. To achieve this, we conducted 16S ribosomal RNA amplicon sequencing (16S rRNA, microbiota profiling) on the rumen fluid samples obtained from the various treatment groups. Notably, we found that Prevotella, Rikenellaceae_RC9_gut group, F082, Muribaculaceae, Christensenellaceae_R-7group, [Eubacterium]_coprostanoligenes_group, Prevotellaceae_UCG-003, Prevotellaceae_UCG-005, Treponema, NK4A214_group, p-251-05, Saccharofermentans, Prevotellaceae_UCG-010, Ruminococcus, and Fibrobacter showed significant enrichment, with Fibrobacter, Ruminococcus, Prevotella, and Muribaculaceae being the dominant genera across the four groups through statistical significance analysis of the A/B tests (Figure 2A,B). The treatment with Lactobacillus significantly reduced the relative abundance of Prevotella and Ruminococcus, while simultaneously increasing the relative abundance of Fibrobacter and Muribaculaceae within the rumen microbiota (Kruskal–Wallis H-test, *p* < 0.05, Figure 3). This finding indicated that the Lactobacillus used in this study enhanced the presence of beneficial bacteria involved in nutrient digestion and absorption. Furthermore, the administration of a high-energy diet with the Lactobacillus supplement (HLG) resulted in a greater number of operational taxonomic units (OTUs) in the rumen microbiota compared to the high-energy diet group (HEG). The rumen microbiota structure, particularly the microbial community related to nutrient digestion and absorption, exhibited a relatively active state, aligning more closely with that of the low-energy diet group (LEG). This suggested that we were able to identify the community changes associated with the effect of HED on the most relevant OTUs through the Lactobacillus supplementation strategy (Figure 4). Consequently, an intervention involving Lactobacillus may play a significant role in preventing rumen microbiota disruption.

**Figure 5:**
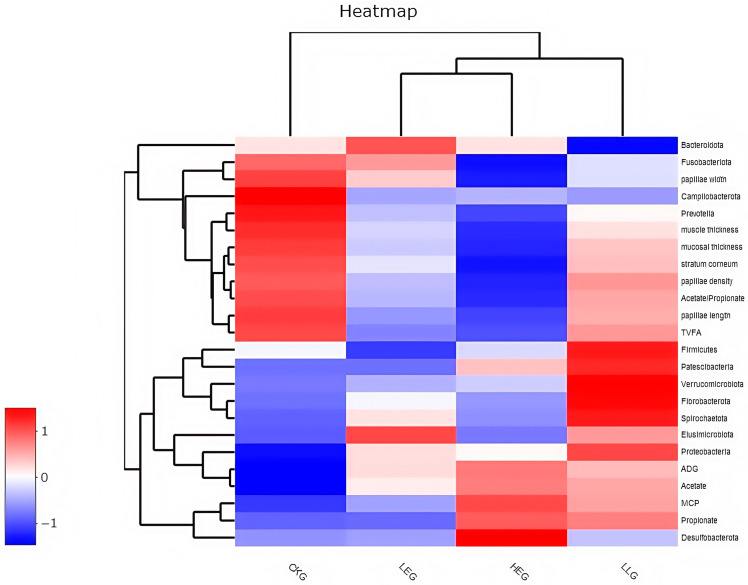
Correlation between the differential treatments and phenotypic traits of the rumen for the yak diets of CKG (0.11 MJ/kg NEG), LEG (2.12 MJ/kg NEG), HEG (2.69 MJ/kg NEG), and HLG (2.69 MJ/kg NEG + 0.02% Lactobacillus). Each row in the graph represents a treatment, each column represents a phenotypic trait, and each lattice represents a Spearman correlation coefficient between a treatment and a morphological trait. Red represents a positive correlation, while blue represents a negative correlation.

Subsequently, Pearson correlation was employed to analyze the correlation among the rumen microbiota, rumen fermentation parameters, and epithelial morphology, and Spearman correlation was utilized to analyze the correlation between the rumen microbiota, rumen fermentation parameters, epithelial morphology, and the different treatments (Figure 5). The rumen microbiota, rumen fermentation parameters, and epithelial morphology of the different treatments were directly subjected to hierarchical clustering, resulting in a pattern consisting of two major sample clusters. The energy diets of the treatment groups shared similar patterns, with the low-energy diet and high-energy diet groups forming one sub-cluster and the high-energy diet with Lactobacillus supplementation group joining this group to constitute another sub-cluster. The control group constituted another sub-cluster. Although the different treatment groups had the same differential expression patterns of rumen microbiota, rumen fermentation parameters, and epithelial morphology, hierarchical clustering analysis revealed differences in different energy diets and Lactobacillus supplementation. These differentially expressed proteins are clearly presented in the hierarchical clustering map produced by view tree software (version 2.0.8) in Figure 5. The signature microbiota components of the different treatments, such as Proteobacteria, Fusobacteriota, Gemmatimonadota, Bdellovibrionota, Campylobacterota, Prevotella, and Firmicutes, were negatively correlated with TVFA, acetate/propionate, and epithelial morphology, while Synergistota, Deferribacterota, and Spirochaetota were positively correlated with TVFA, acetate/propionate, and epithelial morphology (*p* < 0.05).

**Figure 6:**
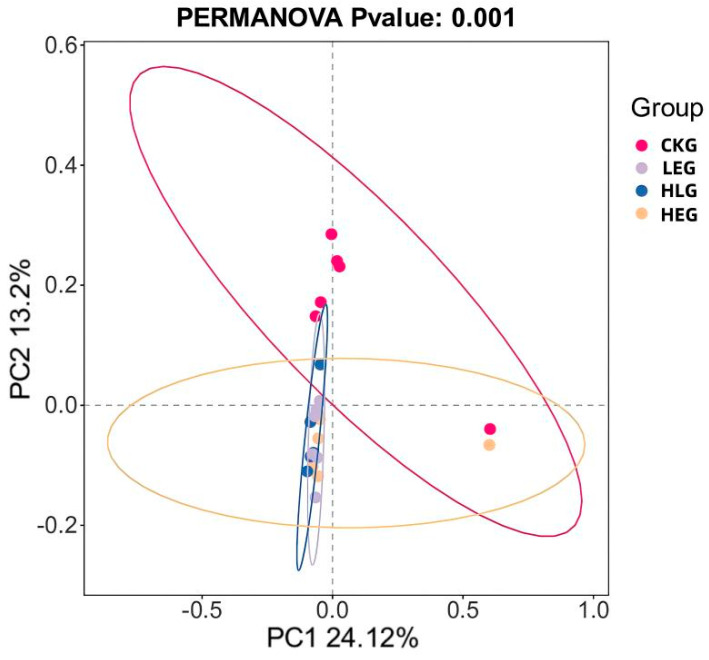
Bacterial PCoA analysis of ruminal microbiota when feeding for the yak diets of CKG (0.11 MJ/kg NEG), LEG (2.12 MJ/kg NEG), HEG (2.69 MJ/kg NEG), and HLG (2.69 MJ/kg NEG + 0.02% Lactobacillus).

The assessment of alpha diversity was carried out by employing the diversity indices (Shannon and Simpson) in conjunction with the richness estimators (Chao1 and ACE). As indicated in Table 5, the richness estimators (ACE and Chao1) rose for LEG, HEG, and HLG in contrast to CKG. Nevertheless, the Shannon index was significantly lower than that of CKG (*p* < 0.05), while the Simpson index was heightened compared to CKG. Notably, HLG manifested a greater vitality in relation to HEG. These findings imply that the addition of Lactobacillus might enhance the richness and diversity of rumen microbiota, especially in the context of Lactobacillus intervention during a high-energy diet mediation. Furthermore, differences in the microbial beta-diversity among the various groups were evaluated using the weighted UniFrac distance (Figure 6). The LEG and HEG displayed higher beta-diversity of the gut microbiota, suggesting a tendency towards fragile microbiota stability and a broader distribution. Additionally, the beta-diversity observed in HLG was lower than that in LEG and HEG, indicating a beneficial effect on rumen microorganisms affected by high-energy diet mediation due to Lactobacillus supplementation. Principal coordinates analysis (PCoA) using the unweighted UniFrac similarity method disclosed that PC1 and PC2 accounted for 24.12% and 13.2% of the total variance among the samples, respectively. This analysis demonstrated that rumen fluid samples from distinct groups formed separate clusters in the ordination space, with the microbiota composition showing more closely arranged clusters and divergence following different energy diet mediation and Lactobacillus intervention as compared to samples from CKG. The microbiota composition exhibited increased divergence within the Lactobacillus treatment group as well as between the different energy diets and Lactobacillus supplementation.

### 3.3. Identification of HED and Lactobacillus Intervention Treatment Markers by Demonstrating Variations in Kyoto Encyclopedia of Genes and Genomes (KEGG) Orthology (KO)

**Figure 7:**
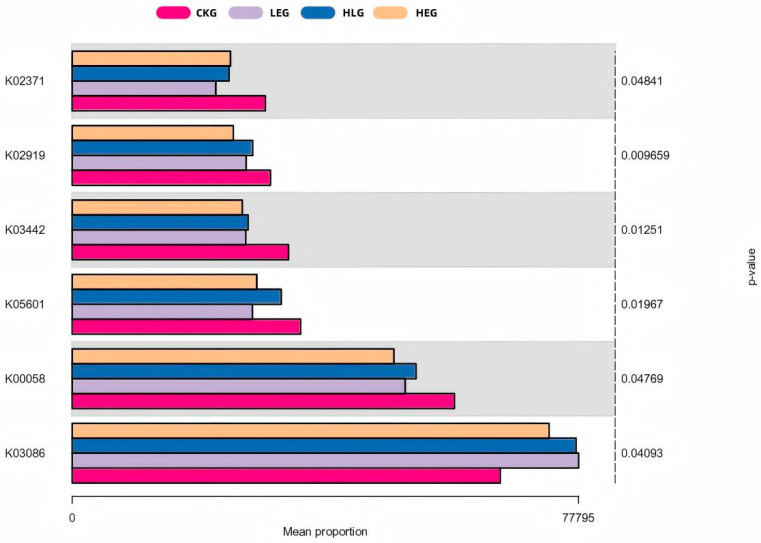
Predicted rumen microbial functions using enrichment KEGG analysis for the yak diets of CKG (0.11 MJ/kg NEG), LEG (2.12 MJ/kg NEG), HEG (2.69 MJ/kg NEG), and HLG (2.69 MJ/kg NEG + 0.02% Lactobacillus).

The analysis of 16S rRNA function plays a crucial role in predicting the complex interactions between the rumen microbiome and livestock health. In our study, we annotated microbiome families obtained from 16S rRNA sequencing as KO abundances using the KEGG orthology database, resulting in an initial set of 5904 KO abundances. After applying a low-abundance filtering step, we obtained a final dataset of 1827 KO abundances for differential abundance analysis. To avoid overfitting, we used a significance analysis of the A/B test (*p* < 0.01), which identified the top 50 differentially expressed KO abundances across different treatment groups (Figure 7). Most of these KO abundances were associated with amino acid and fatty acid metabolism pathways, particularly those involved in fatty acid and protein synthesis. This indicated a strong association between the rumen microbiota and their metabolic activities.

**Figure 8:**
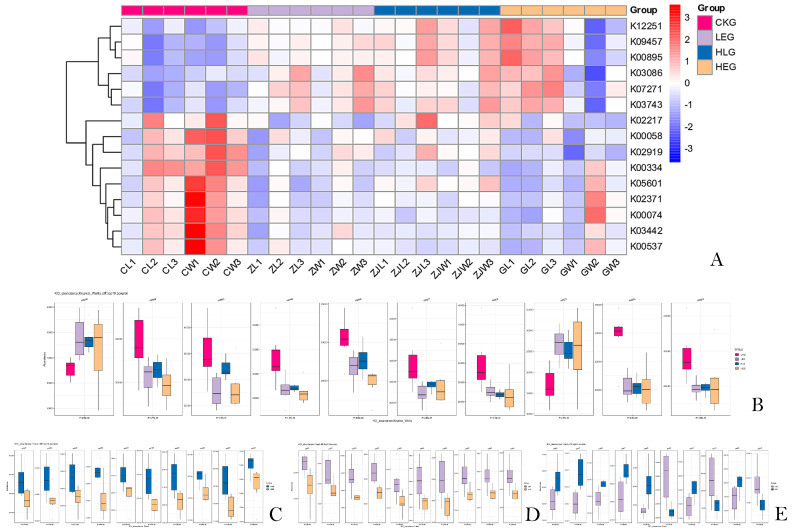
Bacterial KEGG analysis of the KO abundance terms of the ruminal microbiota of the yaks in the diet groups of CKG (0.11 MJ/kg NEG), LEG (2.12 MJ/kg NEG), HEG (2.69 MJ/kg NEG), and HLG (2.69 MJ/kg NEG + 0.02% Lactobacillus). (**A**) The top 10 pathways with potential implications; (**B**) the enriched KEGG terms and functions with different treatments; (**C**–**E**) the enriched KEGG terms and functions with each two treatments.

Subsequently, an enrichment analysis was conducted on these KO abundance terms, revealing the top 10 pathways with potential implications for both traditional grazing groups and HED-mediated treatment groups (*p* < 0.05). Seven of the targeted pathways in the HED-mediated treatment groups showed downregulated expression (Figure 8A), including “Glycine, serine and threonine metabolism”, “Nitrogen metabolism”, “Ribosome”, “Fatty acid biosynthesis”, “Arsenate reductase (glutaredoxin)”, “Oxidative phosphorylation”, and “Phenylalanine metabolism.” Further analysis (CKG, LEG, HLG, and HEG) revealed that “Ribosome” (K02919) and “Fatty acid biosynthesis” (K02371) played vital roles in the synthesis and regulation of short-chain fatty acids in certain rumen microbiota (Figure 8B). “Glycine, serine and threonine metabolism” (K00058), “Ribosome” (K02919), “Fatty acid biosynthesis” (K02371), “Nitrogen metabolism” (K05601), “Amino sugar and nucleotide sugar metabolism” (K02564), “Apoptosis” (K03386), “Ferritin” (K02217), “Thiamine metabolism” (K03149), “Arsenate reductase (glutaredoxin)” (K00537), “Oxidative phosphorylation” (K00334), and “Phenylalanine metabolism” (K00074) were identified as the most enriched KO abundances with the highest generalized fold changes in the Lactobacillus intervention treatment. Additionally, several pathways, such as K00058, K05601, K02919, and K02371, were upregulated in HLG compared to HEG, indicating a positive correlation with Lactobacillus supplementation (Figure 8C–E). It has been reported that fatty-acid-metabolism-related pathways (K00058, K05601, and K02371), protein-synthesis-related pathways (K02919, K02217, K02564, and K00074), and other metabolism-related pathways (K03386, K00537, and K00334) enhance the development of the rumen barrier structure and functional integrity through Lactobacillus-mediated mechanisms, playing a critical role in resisting SCFA intrusion processes induced by HED and promoting rapid regeneration of the rumen mucosa.

### 3.4. Demonstration of Metabolomic Alterations Across Different Lactobacillus Supplementation Strategies and Utilization of Signature Metabolites for HED-Mediated Rumen Barrier Dysfunction

**Figure 9:**
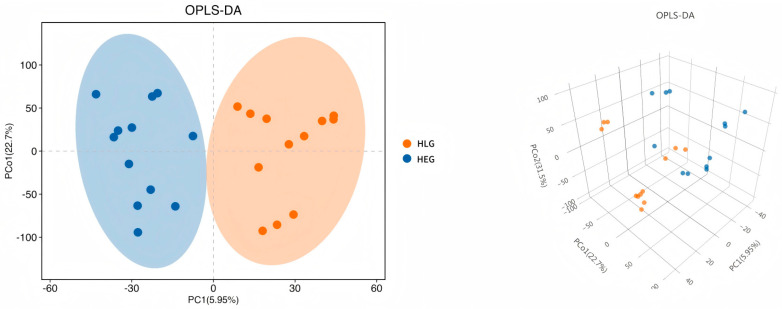
Plot of the 3D-PCA and OPLS-DA scores, and a volcano plot of the ruminal metabolites for the yak diets of HEG (2.69 MJ/kg NEG) and HLG (2.69 MJ/kg NEG + 0.02% Lactobacillus).

**Figure 10:**
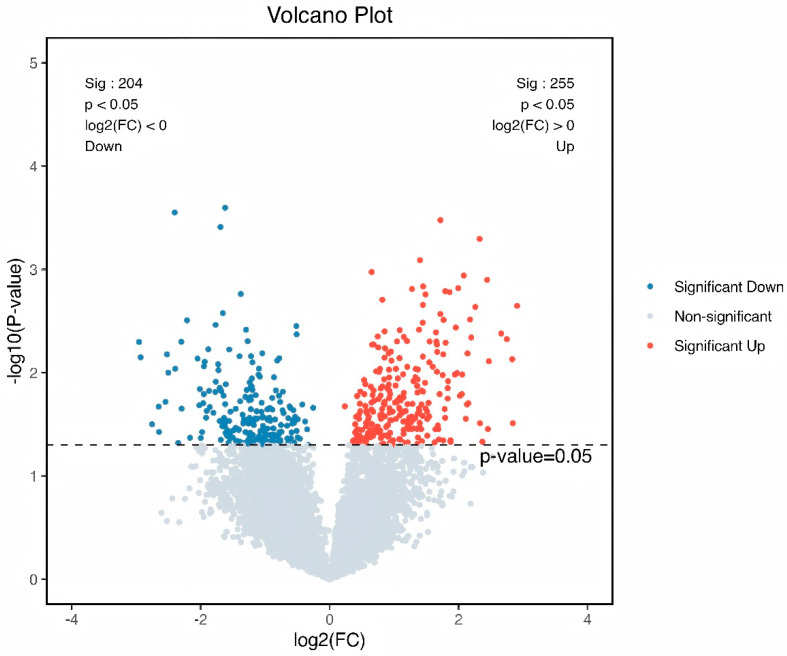
Volcano plot analysis for the identification of different metabolites from the yaks in the diet groups of HEG (2.69 MJ/kg NEG) and HLG (2.69 MJ/kg NEG + 0.02% Lactobacillus). Each point in the figure represents a sample, where red indicates that the metabolite is expressed at high levels, and blue indicates lower expression.

**Figure 11:**
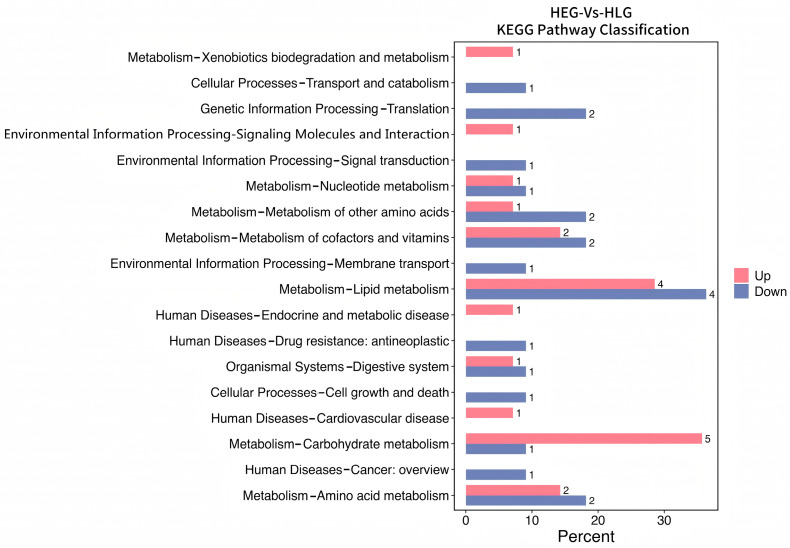
Metabolic KEGG pathway enrichment analysis following positive and negative mode ionization to provide a metabolite overview for the yaks in the diet groups of HEG (2.69 MJ/kg NEG) and HLG (2.69 MJ/kg NEG + 0.02% Lactobacillus).

Intrigued by the intricate interplay between rumen microbiota and host co-metabolism, we further explored the spectrum of changes in ruminal metabolites through metabolomics. Using 3D-PCA and OPLS-DA multivariate statistical analysis models, we evaluated the differences between the high-fat diet (HEG) and the Lactobacillus intervention treatment (HLG) via score plots (Figure 9). The 3D-PCA score plots derived from the LC-TOF/MS ruminal fluid metabolic profiles demonstrated a clear separation between HEG and HLG. A distinct separation was observed with Lactobacillus supplementation, indicating that the OPLS-DA model could effectively identify differential metabolites in the yak rumen between HEG and HLG. To further elucidate the differences in the metabolites for the yak rumen under high-fat diet conditions with Lactobacillus intervention, we conducted a differential analysis using volcano plots (Figure 10) and identified 50 metabolites (Table S1). The majority of these differentially abundant metabolites belonged to three major nutrient metabolism categories: amino acids, carbohydrates, and fatty acids. Compared with the yaks on a high-fat diet, 37 metabolites were increased and 13 metabolites were decreased in yaks subjected to the Lactobacillus intervention. These results, from the analysis of the rumen of the Pamir yaks, were involved in multiple biochemical processes, including “Lipid metabolism”, “Carbohydrate metabolism”, “Cell growth and death”, “Translation”, “Amino acid metabolism”, and “Metabolism of other amino acids (Figure 11).

**Figure 12:**
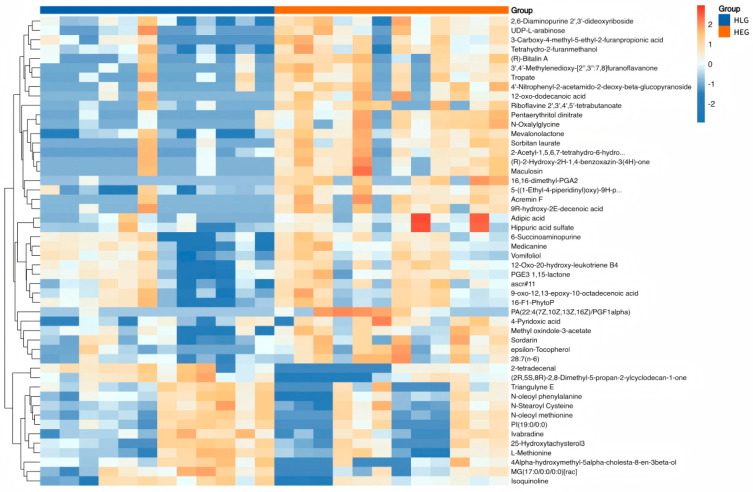
Hierarchical clustering analysis for the identification of different metabolites in the yaks of the diet groups HEG (2.69 MJ/kg NEG) and HLG (2.69 MJ/kg NEG + 0.02% Lactobacillus). Each column in the figure represents a sample, each row represents a metabolite, and the color indicates the relative amounts of the metabolites expressed in the group; red indicates that the metabolite is expressed at high levels, and blue indicates lower expression.

**Figure 13:**
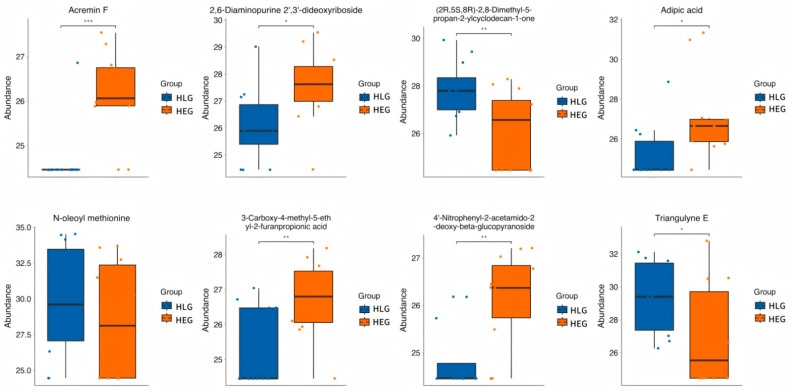
Student’s *t* test analysis of the metabolic enrichment pathways following positive and negative mode ionization to provide a metabolite overview for the yaks in the diet groups of HEG (2.69 MJ/kg NEG) and HLG (2.69 MJ/kg NEG + 0.02% Lactobacillus). * Significant correlation between HLG and HEG (*p* < 0.05), ** Significant correlation between HLG and HEG (*p* < 0.01), *** Significant correlation between HLG and HEG (*p* < 0.001).

Hierarchical clustering analysis (HCA) with a heatmap was performed to visualize the differences in the yak rumen metabolome associated with HEG and HLG. The hierarchical clustering clearly demonstrated similar clustering patterns of the molecular features within each treatment group. Lactobacillus supplementation significantly impacted the HED-mediated rumen metabolome (*p* < 0.05), and cluster differences were clearly observable in the HCA-generated heatmap. Interestingly, metabolic pathway enrichment analysis revealed that various metabolites related to fatty acid biosynthesis (Figure 12), such as 2-tetradecenal, Triangulyne E, adipic acid, (2R,5S,8R)-2,8-dimethyl-5-propan-2-ylcyclodecan-1-one, epsilon-tocopherol, 3-carboxy-4-methyl-5-ethyl-2-furanpropionic acid, 16,16-dimethyl-PGA2, sorbitan laurate, Acremin F, 5-((1-Ethyl-4-piperidinyl)oxy)-9H-pyrrolo(2,3-b:5,4-c’)dipyridine-6-carbonitrile, 4′-nitrophenyl-2-acetamido-2-deoxy-beta-glucopyranoside, tetrahydro-2-furanmethanol, N-oleoyl methionine, 3′,4′-methylenedioxy-[2″,3″:7,8]furanoflavanone, 9-oxo-12,13-epoxy-10-octadecenoic acid, 2,6-diaminopurine 2′,3′-dideoxyriboside, pentaerythritol dinitrate, and mevalonolactone were enriched by the Lactobacillus intervention treatment (*p* < 0.05; rich factor > 0.10). This suggested the fatty acid metabolism of ruminal microbiota under Lactobacillus intervention tended to be normal. Consistent with PICRUSt function prediction pathways, fatty acid metabolism was significantly enriched in yaks subjected to Lactobacillus intervention compared with those on a high-fat diet. The boxplot value for each metabolite was calculated, and the metabolites with significant *p*-values of *p* < 0.05 and *p* < 0.01 were selected as potential biomarkers. Eight metabolites identified through the Student’s *t*-test (Figure 13) were as follows: Acremin F, 2,6-diaminopurine 2′,3′-dideoxyriboside, (2R,5S,8R)-2,8-dimethyl-5-propan-2-ylcyclodecan-1-one, adipic acid, N-oleoyl methionine, 3-carboxy-4-methyl-5-ethyl-2-furanpropionic acid, 4′-nitrophenyl-2-acetamido-2-deoxy-beta-glucopyranoside, and Triangulyne E.

### 3.5. Multi-Omics Signature Integration for HED and Lactobacillus Intervention Treatment

**Figure 14:**
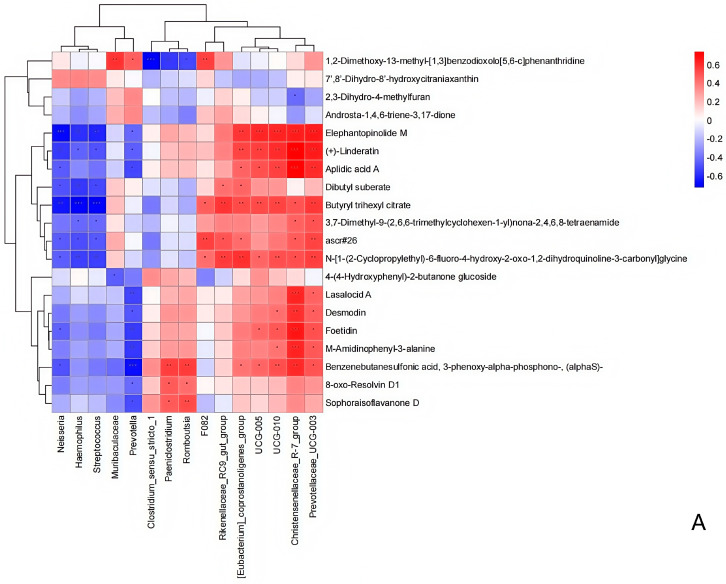

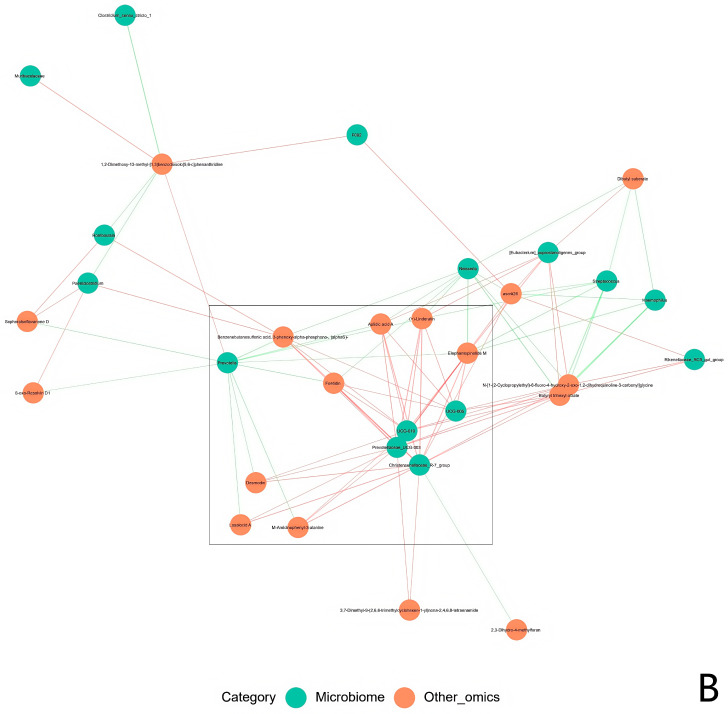
Correlation analysis between the ruminal microbiota and metabolite concentrations (VIP > 2) in the yaks of the diet groups HEG (2.69 MJ/kg NEG) and HLG (2.69 MJ/kg NEG + 0.02% Lactobacillus). (**A**). Each row in the graph represents a metabolite, each column represents a ruminal microbiota, and each lattice represents a Pearson correlation coefficient between a component and a metabolite. Red represents a positive correlation, while blue represents a negative correlation. * Significant correlation between HLG and HEG (*p* < 0.05), ** Significant correlation between HLG and HEG (*p* < 0.01), *** Significant correlation between HLG and HEG (*p* < 0.001). (**B**). The interaction network between microorganisms (genus level) and metabolites is presented. The boxe denote the aggregation status of target microorganisms and metabolites.

The functional correlation between the ruminal microbiome changes and metabolite perturbations (VIP > 2, *p* < 0.05) was assessed using a correlation matrix generated by calculating the Spearman correlation coefficient. Significant correlations were identified between the perturbed ruminal microbiota and altered metabolite profiles (r > 0.5 or r < −0.5, *p* < 0.05). Specifically, Fibrobacter exhibited a negative correlation with 2,6-diaminopurine 2′,3′-dideoxyriboside; Prevotella and Ruminococcus showed positive correlations with (2R,5S,8R)-2,8-dimethyl-5-propan-2-ylcyclodecan-1-one, epsilon-Tocopherol, 3-carboxy-4-methyl-5-ethyl-2-furanpropionic acid, 16,16-dimethyl-PGA2, tetrahydro-2-furanmethanol, N-oleoyl methionine, 3′,4′-methylenedioxy-[2″,3″:7,8]furanoflavanone, 9-oxo-12,13-epoxy-10-octadecenoic acid, 2,6-diaminopurine 2′,3′-dideoxyriboside, and pentaerythritol dinitrate, while displaying negative correlations with Triangulyne E, adipic acid, sorbitan laurate, 5-((1-ethyl-4-piperidinyl)oxy)-9H-pyrrolo(2,3-b:5,4-c’)dipyridine-6-carbonitrile, and mevalonolactone. Muribaculaceae demonstrated a negative correlation with 2-tetradecenal, tetrahydro-2-furanmethanol, 9-oxo-12,13-epoxy-10-octadecenoic acid, 2,6-diaminopurine 2′,3′-dideoxyriboside, and pentaerythritol dinitrate (Figure 14). In summary, the HED-mediated processes induced taxonomic perturbations in the yak ruminal microbiome, which, in conjunction with the Lactobacillus intervention treatment, substantially altered the ruminal metabolomic profile due to changes in diverse ruminal microbiota-related metabolites.

## 4. Discussion

The yak is a unique domestic animal species specifically adapted to the plateau regions, which is predominantly reared in an open system and thus more susceptible to external environmental influences under traditional feeding practices [29]. Extreme climate variability and nutritional deficiencies are key factors leading to reduced productivity and increased disease susceptibility in yaks [30]. To enhance production efficiency, livestock farmers often shift the feeding regimen toward high-energy or high-fat diets [31]. However, accumulating evidence has revealed that such dietary changes may exacerbate the gastrointestinal metabolic load in yaks and compromise their health and productive potentialities [32]. Our study offers new insights into the dynamic interplay between ruminal microbiota remodeling and metabolic reprogramming in response to high-energy diet (HED) feeding and Lactobacillus intervention. By integrating 16S rRNA sequencing, metabolomics, and phenotypic trait modeling, we systematically characterized the taxonomic shifts in key rumen microbiota (e.g., Prevotella, Ruminococcus, Fibrobacter, and Muribaculaceae) and elucidated the HED-induced dysregulation of amino acid and fatty acid metabolism pathways (K02371, K02919). Notably, Lactobacillus supplementation not only mitigated HED-mediated microbial colonization but also restored 44 metabolites associated with amino acid metabolism, fatty acid conjugation, and carbon-nitrogen recycling, thereby extending prior findings on probiotic-mediated rumen homeostasis. Compared to earlier single-omics studies in ruminants, our multi-omics approach achieved superior diagnostic precision for identifying HED-mediated microbiota. Furthermore, the functional enrichment of Prevotella-associated fatty acid metabolism and Ruminococcus-driven fatty acid synthesis challenges the conventional view of strict substrate-specific microbial niches.

The rumen is the primary organ responsible for dietary nutrient digestion, absorption, and metabolism, playing a critical role in yak health [33]. Any alterations or dysfunction in the ruminal architecture can adversely affect feed efficiency, overall productivity, and animal health [34]. Previous studies have demonstrated that adjusting dietary energy levels can enhance the body weight of yaks and their performance in the yak industry [35,36]. In our study, yaks in the high-energy group (HEG) exhibited greater weight gain with a higher average daily gain compared to both the control group (CKG) and low-energy group (LEG). The results showed that a commercial strain of Lactobacillus (Lactobacillus fermentum), with an effective Lactobacillus content of 2.4 billion CFU, could be used, and the yaks receiving the Lactobacillus intervention showed faster body weight growth compared to those fed only with high-energy diets (HED). This indicated that different energy diets can improve the yak’s growth performance to varying extents. Lactobacillus exhibits significant potential as an effective feed additive for re-engineering microbiota function and metabolism and enhancing production efficiency, which are findings consistent with earlier studies [37]. Volatile fatty acids (VFAs) are the products of rumen microbial degradation of substances such as cellulose, pentosans, and proteins, providing 70–80% of the energy requirements for ruminants [38]. To our knowledge, VFAs are primarily derived from carbohydrate hydrolysis mediated by ruminal microbes, and dietary energy levels can significantly influence VFA production. Acetic acid serves as a precursor for fat synthesis in ruminants, while propionic acid plays a critical role in hepatic glycogen synthesis. With the addition of dietary energy, the concentrations of TVFAs, acetic acid, butyric acid, and MCP were significantly increased. These results indicated that elevated concentrations of TVFAs, acetic acid, butyric acid, and MCP in the rumen reflect improved energy conversion efficiency, which is essential for animal weight gain. Changes in the molar proportions of acetate/propionate revealed shifts in VFA-producing pathways and microbial populations, while the concentrations of propionic and butyric acids exhibited strong correlations with dietary energy levels and Lactobacillus supplementation [39]. Lactobacillus supplementation enhances digestive gut enzyme activity and regulates rumen energy homeostasis by optimizing nutrient extraction from the diet to maximize the potential economic benefits. In this study, the high-Lactobacillus group (HLG) exhibited a lower acetate/propionate ratio compared to the high-energy group (HEG), yet the yaks showed enhanced body weight growth performance to some extent. The morphophysiological variations in the ruminal epithelium of the ruminants reflect their capacity for dietary nutrient digestion and absorption [40]. In this study, we observed that the high-energy diets led to a reduction in the rumen tissue morphology parameters (stratum corneum thickness, tunica propria thickness, mucosal epithelial thickness, submucosal thickness, muscle layer thickness, epithelial papillae density, epithelial papillae length, and epithelial papillae width), thereby decreasing the selective absorption capacity of nutrients in the yak rumen. Changes in the structure of the villous absorptive surface (height, width, and crypt depth) in the ruminal epithelium were closely associated with rumen health status. Specifically, as the rumen tissue morphology improved, the ability to digest and absorb dietary nutrients increased, leading to elevated carbohydrate levels and enhanced rumen microbial fermentation. When Lactobacillus supplementation was provided alongside a high-energy diet, it reduced the concentration of free short-chain fatty acids (SCFAs) produced by the microbial degradation of nutrients in the rumen, which in turn decreased gut volatile fatty acids (VFAs) and altered the gut pH values.

Rumen microbiota play an indispensable role in degrading complex nutritional components, such as dietary energy, and modulating the host’s metabolism of bile acids, lipids, and amino acids [41]. Acting as a “secondary genome” that influences the health characteristics of the host superorganism, ruminal microbiota are closely linked to host nutrition, metabolic processes, and immune responses. Bacteria are the most prevalent and diverse microorganisms in the rumen, assisting in diet fermentation and producing nutrients for the host [42]. In this study, we investigated the impact of a high-energy diet (HED) and Lactobacillus supplementation on the yak rumen microbiota structure and function through 16S rRNA high-throughput sequencing. Venn diagram analysis revealed that 20 phyla were shared across all four groups, indicating that the core microbiota remained stable. Richness estimators (ACE and Chao1) increased in the HLG group compared to the HEG group, whereas the Shannon index significantly decreased, suggesting that a high-energy diet increased microbial diversity but reduced the evenness of microbial communities, particularly in the Lactobacillus-supplemented samples. This study identified Fibrobacter, Ruminococcus, Prevotella, and Muribaculaceae as the dominant genera across all treatments, collectively constituting over 90% of the rumen microbiota. Notably, the rumen microbiota composition in the Lactobacillus-treated yaks resembled that of yaks on a low-energy diet. Thus, our findings suggested that Lactobacillus supplementation can partially restore gut microbiota imbalances induced by a high-fat diet, reducing the relative abundance of Prevotella and Ruminococcus while increasing the relative abundance of Fibrobacter and Muribaculaceae. One potential explanation for these findings is the negative correlation between Fibrobacter and lipid-soluble nutrient metabolism, contrasted with the positive correlation observed for Firmicutes. This implies that Lactobacillus intervention may enhance the lipid metabolism of dietary nutrients. Functional analysis of the rumen microbiota revealed that most KOs were associated with amino acid and fatty acid metabolism pathways, particularly those involved in fatty acid and protein synthesis. Fatty-acid-metabolism-related pathways (K00058, K05601, and K02371), protein-synthesis-related pathways (K02919, K02217, K02564, and K00074), and other metabolism-related pathways (K03386, K00537, and K00334) contributed to the development of the rumen barrier structure and functional integrity via Lactobacillus-mediated mechanisms, resisting short-chain fatty acid (SCFA) intrusion induced by HED and promoting rapid regeneration of the rumen mucosa. Significant variations in the rumen microbial composition were observed in association with different food sources and geographic settings, highlighting the need to establish correlations between compositional and functional variances in the rumen microbiota. Dysfunctional states within the microbiome are typically characterized by compositional alterations, particularly the depletion of beneficial microbes. Lactobacillus supplementation can elevate levels of gut barrier-protecting bacteria and inhibit the growth of endotoxin-producing organisms. Consequently, we inferred that the selective modulation of dietary gut microbial phylotypes via Lactobacillus supplementation may mitigate host gut microbiota dysbiosis induced by a high-fat diet.

Alterations in the metabolite profiles of microbial communities can reflect dynamic changes within these communities [43]. Therefore, defining the relationships between metabolic functions and microbial community structures through correlation analyses may provide valuable insights into a comprehensive understanding of the microbial composition and community function. Lactobacillus, as a probiotic, can protect the rumen bacterial environment from harmful dietary components. Some studies have demonstrated that supplementation with Lactobacillus may enhance diet nutrient availability and elevate the metabolic activity of rumen microbiota, which is associated with increased weight gain in yaks [44,45]. The metabolomics results revealed that the Lactobacillus intervention treatment altered ruminal metabolite concentrations, suggesting a potential link between ruminal metabolism and ruminal microbiota activity. Lactobacillus supplementation altered the concentrations of multiple metabolites associated with nutrient digestion and absorption in yaks. The metabolites related to fatty acid biosynthesis concentration in HLG was higher than that in the yak rumen from CNK. These target amino acids play a critical role in regulating rumen mucosal antigen responses, which may contribute to improving intestinal barrier function by enhancing fatty acid metabolism and protein synthesis pathways. This effect could potentially reduce the content of free SCFAs in the rumen and improve ruminal barrier integrity. Our studies found eight target metabolites (Acremin F, 2,6-diaminopurine 2′,3′-dideoxyriboside, (2R,5S,8R)-2,8-dimethyl-5-propan-2-ylcyclodecan-1-one, adipic acid, N-oleoyl methionine, 3-carboxy-4-methyl-5-ethyl-2-furanpropionic acid, 4′-nitrophenyl-2-acetamido-2-deoxy-beta-glucopyranoside, and Triangulyne E), which were related to fatty acid metabolism and anti-inflammatory regulation. The metabolites 3-carboxy-4-methyl-5-ethyl-2-furanpropionic acid and Triangulyne E protect the rumen mucosa through their antioxidant effects. Meanwhile, Acremin F, (2R,5S,8R)-2,8-dimethyl-5-propan-2-ylcyclodecan-1-one, adipic acid, N-oleoyl methionine, and 4′-nitrophenyl-2-acetamido-2-deoxy-beta-glucopyranoside may jointly alleviate the rumen barrier function impairment induced by high-energy feed by regulating the lipid metabolism balance and inflammatory responses [46]. The present study demonstrated that Lactobacillus intervention treatment can optimize the composition, function, diversity, and activity of ruminal microbiota, thereby reducing the need for dietary energy supplementation and feed costs while maintaining host health through the altering of metabolite availability. Spearman correlation analysis indicated that feeding a high-energy diet supplemented with Lactobacillus induced changes in the ruminal microbial community and metabolite functions, leading to shifts in phenotypic traits; for instance, ruminal epithelial tissue morphology and fermentation parameters exhibited tendencies toward those observed in CNK and LEG. In addition to influencing the ruminal microbial types, numbers, metabolism, and physiology, the supplementation of Lactobacillus may regulate other mechanisms, such as modulating the bacterial gene and protein expression; for example, acetate and butyrate production by the ruminal microbiome mediates the regulation of growth-related genes in the rumen epithelium, which in turn regulates various epithelial physiological processes [47]. These studies, along with ours, have demonstrated that Lactobacillus intervention treatment alters the composition of the ruminal microbiome and their associated metabolic profiles in Pamir Yaks. Therefore, it is reasonable to think that differences in ruminal structure and function result from the diet, specifically Lactobacillus intervention, host microbe–metabolite interactions, and/or the host’s physiological state.

## 5. Conclusions

These data clearly demonstrated that high-energy diet feeding may impair yak ruminal histomorphology and their microbiota composition and function, while negatively modulating the metabolic profiles associated with specific ruminal microbial communities and functions. Lactobacillus intervention treatment optimized the yak ruminal microbiome composition, thereby altering metabolite concentrations involved in various metabolic pathways under a high-energy feeding pattern. These alterations helped elucidate the beneficial impacts of the Lactobacillus supplementation strategy on yak ruminal health without compromising the high-energy intensive rearing pattern. Furthermore, the regulated ruminal microbiome metabolites may serve as potential biomarkers for future investigations into the functional impacts of Lactobacillus intervention treatment on healthy feeding strategies for yaks. These findings support incorporating Lactobacillus supplementation in the commercial fattening diets for yaks.

## Figures and Tables

**Table 1:** The feed ingredients and nutrient composition for the yak diets of CKG (0.11 MJ/kg NEG), LEG (2.12 MJ/kg NEG), and HEG (2.69 MJ/kg NEG).

Composition (%)	CKG	LEG	HEG
Maize	0	29.8	34.4
Cottonseed meal	0	4.6	5
Soybean meal	0	6.0	6.5
Soybean oil	0	0	0.6
Wheat bran	0	5.8	1
Molasses	0	1.3	0
Premix ②	0	2.5	2.5
Wheat straw	0	50	50
Native grass	100	0	0
Total	100	100	100
Nutritional composition ① ③			
CP (%)	2.64	9.85	10.92
NEG (MJ/Kg)	0.11	2.12	2.69
ADF (%)	16.79	5.18	4.51
NDF (%)	55.05	43.42	41.84
Ca	0.18	1.62	1.59
P	0.05	0.55	0.54

Note: ① The values for NEG are the calculated values (NRC, 2007 [28]), the other values are measured values. ② The premix provided the following, per kg of the diets: Fe 2500 mg, Zn 1000 mg, Cu 1000 mg, Mn 1000 mg, Se 7.5 mg, I 20 mg, VA 300,000 IU, VD 5000 IU, VE 4000 IU. ③ CP is Crude Protein, NEG is Net Energy, ADF is Acid Detergent Fiber, NDF is Neutral Detergent Fiber, Ca is Calcium, P is Phosphorus.

**Table 2:** The body weight (BW) and average daily gain (ADG) for the yak diets of CKG (0.11 MJ/kg NEG), LEG (2.12 MJ/kg NEG), HEG (2.69 MJ/kg NEG), and HLG (2.69 MJ/kg NEG + 0.02% Lactobacillus).

Groups	Initial Body Weight (kg)	Final Body Weight (kg)	Total Gain (kg)	Average Daily Gain (g/d)
CKG	260.15 ± 35.55	279.40 ± 16.80 d	19.25 ± 10.15 c	113.24 ± 48.90 c
LEG	257.45 ± 27.25	317.25 ± 23.50 c	59.80 ± 21.70 b	351.76 ± 98.95 b
HEG	255.35 ± 25.35	349.85 ± 43.45 b	94.50 ± 21.25 a	555.88 ± 113.65 ab
HLG	264.30 ± 29.20	367.85 ± 35.20 a	103.55 ± 21.55 a	609.12 ± 102.85 a

Note. Different letters represent significant differences (*p* < 0.05).

**Table 3:** The ruminal fermentation parameters (VFAs) for the yak diets of CKG (0.11 MJ/kg NEG), LEG (2.12 MJ/kg NEG), HEG (2.69 MJ/kg NEG), and HLG (2.69 MJ/kg NEG + 0.02% Lactobacillus).

Groups	Acetic Acid (mmol/L)	Propanoic Acid (mmol/L)	Isobutyric Acid (mmol/L)	Butyric Acid (mmol/L)	Isovaleric Acid (mmol/L)	Valeric Acid (mmol/L)	pH	NH3-N (mg/dL)	TVFA (mmol/L)	MCP (g/L)	A/P
CKG	39.93 ± 2.25 b	25.33 ± 1.31	2.11 ± 0.09	22.19 ± 0.36	1.56 ± 0.04 b	3.23 ± 0.08	6.81 ± 0.01 a	26.19 ± 0.11 a	95.66 ± 1.07 d	1.92 ± 0.01 a	1.63 ± 0.09 a
LEG	43.84 ± 1.37 ab	25.39 ± 1.54	2.17 ± 0.08	22.88 ± 0.31	1.61 ± 0.05 b	3.35 ± 0.11	6.76 ± 0.02 b	24.92 ± 0.07 c	100.54 ± 1.16 c	1.72 ± 0.03 c	1.62 ± 0.15 b
HEG	44.88 ± 1.33 ab	27.77 ± 1.56	2.25 ± 0.05	23.17 ± 0.54	1.69 ± 0.11 b	3.33 ± 0.13	6.51 ± 0.01 c	25.87 ± 0.08 b	104.31 ± 1.23 b	1.85 ± 0.02 b	1.60 ± 0.14 c
HLG	45.48 ± 1.57 a	28.05 ± 1.13	2.30 ± 0.07	22.77 ± 0.38	1.95 ± 0.11 a	3.16 ± 0.11	6.42 ± 0.01 d	24.72 ± 0.07 c	107.04 ± 1.27 a	1.61 ± 0.03 d	1.61 ± 0.13 b

Note. Different letters represent significant differences (*p* < 0.05).

**Table 4:** Rumen tissue morphology for the yak diets of CKG (0.11 MJ/kg NEG), LEG (2.12 MJ/kg NEG), HEG (2.69 MJ/kg NEG), and HLG (2.69 MJ/kg NEG + 0.02% Lactobacillus).

Groups	Stratum Corneum	Tunica Propria	Mucosal Epithelial Thickness	Submucosal Thickness	Muscle Layer Thickness	Epithelial Papillae Density (/mm^2^)	Epithelial Papillae Width (mm)	Epithelial Papillae Length (mm)
CKG	27.76 ± 1.17 a	136.94 ± 6.32 a	102.80 ± 10.37 a	621.45 ± 10.44 a	5364.94 ± 137.14 a	121.18 ± 7.46 a	1.56 ± 0.01 a	5.43 ± 0.18 a
LEG	23.22 ± 1.44 c	121.33 ± 5.21 b	91.05 ± 9.20 b	586.60 ± 10.49 c	4469.45 ± 94.96 c	93.83 ± 7.06 b	1.45 ± 0.01 c	5.05 ± 0.03 b
HEG	18.72 ± 1.20 d	114.13 ± 7.57 c	82.20 ± 10.45 c	564.94 ± 9.50 d	3878.02 ± 135.23 d	75.88 ± 5.49 c	1.42 ± 0.01 c	4.28 ± 0.05 c
HLG	25.28 ± 1.21 b	133.92 ± 6.39 a	98.29 ± 11.18 a	600.85 ± 11.23 b	4733.37 ± 129.83 b	114.21 ± 5.18 a	1.52 ± 0.01 b	4.80 ± 0.05 b

Note. Different letters represent significant differences (*p* < 0.05).

**Table 5:** The alpha diversity of bacterial communities for the yak diets of CKG (0.11 MJ/kg NEG), LEG (2.12 MJ/kg NEG), HEG (2.69 MJ/kg NEG), and HLG (2.69 MJ/kg NEG + 0.02% Lactobacillus).

Groups	ACE	Chao1	Shannon	Simpson
CKG	815.66 ± 7.355 b	819.12 ± 76.33 b	8.91 ± 0.29 a	0.9854 ± 0.01 b
LEG	959.14 ± 27.01 ab	963.32 ± 30.61 ab	8.59 ± 0.07 b	0.9916 ± 0.02 a
HEG	1004.64 ± 50.09 a	1006.22 ± 48.40 a	8.63 ± 0.15 b	0.9944 ± 0.01 ab
HLG	1032.23 ± 43.44 a	1032.82 ± 43.15 a	8.64 ± 0.09 b	0.9940 ± 0.01 a

Note. Different letters represent significant differences (*p* < 0.05).

## Data Availability

The datasets presented in this study can be found in online repositories. All raw sequence data were deposited in the NCBI Sequence Read Archive database under accession number PRJNA1233467.

## Supplementary Materials

The following supporting information can be downloaded at: https://www.mdpi.com/article/10.3390/ani15121681/s1, Table S1. The Top 50 of detailed information of significantly different metabolites at variable importance in projection (VIP) > 1 and *p* < 0.05 (Wilcoxon-test) when feeding Yak diets containing HEG (2.69 MJ/kg NEG) and HLG (2.69 MJ/kg NEG + 0.02% Lactobacillus).
