# Supplementary material for: Lactobacillus Supplementation Modulates Rumen Microbiota and Metabolism in Yaks Under Fattening Feeding Conditions: A Comprehensive Multi-Omics Analysis
Source: Animals (Basel). 2025 Jun 6;15(12):1681. doi: 10.3390/ani15121681 (PMC12190064; doi:10.3390/ani15121681)
Supplement: Supplementary file 1 [file animals-15-01681-s001.zip › animals-3653485-supplementary.pdf]

Table S1. The Top 50 of detailed information of significantly different metabolites at variable importance in projection (VIP) > 1 and P < 0.05 (Wilcoxon-test) when feeding Yak diets containing HEG (2.69 MJ/kg NEG) and HLG (2.69 MJ/kg NEG + 0.02% Lactobacillus).

Metabolite name	Regulation	m/z	RT(s)	Ion mode	FC (HLG/HEG)	P-value
2-tetradecenal	Up	614.3400654	12.60148333	pos	23.37011832	0.003855125
Triangulyne E	Up	634.2633764	8.24285	neg	9.366909164	2.14313E-06
Adipic acid	Up	561.4874961	12.6423	pos	20.40721578	0.010497144
(2R,5S,8R)-2,8-Dimethyl-5-propan-2-ylcyclodecan-1-one	Down	477.3150077	13.50653333	pos	0.057879786	0.009828997
epsilon-Tocopherol	Up	492.3448503	11.2292	pos	9.03540782	0.000100054
3-Carboxy-4-methyl-5-ethyl-2-furanpropionic acid	Up	499.3391765	12.62198333	pos	11.46343765	0.003662598
16,16-dimethyl-PGA2	Up	865.5310834	10.1357	pos	0.042930599	0.049427972
Sorbitan laurate	Down	455.2215848	11.51476667	pos	9.572730269	0.004603751
Acremin F	Up	510.2936077	11.86335	pos	10.42952958	0.006432658
5-((1-Ethyl-4-piperidinyl)oxy)-9H-pyrrolo(2,3-b:5,4-c')dipyridine-6-carbonitrile	Up	510.3557159	13.11586667	pos	11.88833545	0.01105911
4'-Nitrophenyl-2-acetamido-2-deoxy-beta-glucopyranoside	Up	409.2019231	12.10901667	pos	9.121315823	0.007486712
Tetrahydro-2-furanmethanol	Up	362.3030612	13.19655	pos	11.08957784	0.014888784
N-oleoyl methionine	Up	552.3662555	14.27333333	pos	11.13490295	0.025524861
3',4'-Methylenedioxy-[2",3":7,8]furanoflavanone	Up	591.4255212	12.97201667	pos	10.16340781	0.025498493
9-oxo-12,13-epoxy-10-octadecenoic acid	Up	445.3677328	12.02776667	pos	11.01807517	0.018599286
2,6-Diaminopurine 2',3'-dideoxyriboside	Up	473.1961811	10.65891667	pos	8.067483481	0.011461508
Pentaerythritol dinitrate	Up	247.0577269	10.7013	pos	7.852206379	0.009341933
Mevalonolactone	Up	477.1907239	10.5585	pos	10.19270706	0.026680475
ascr#11	Up	510.2841197	11.49513333	pos	12.41717882	0.006064549
28:7(n-6)	Up	572.3689011	13.1769	pos	11.2163099	0.022384531
MG(17:0/0:0/0:0)[rac]	Up	456.2168182	11.35176667	pos	8.367742706	0.013967864
UDP-L-arabinose	Up	454.2785098	11.7197	pos	8.785736576	0.023126182
2-Acetyl-1,5,6,7-tetrahydro-6-hydroxy-7-(hydroxymethyl)-4H-azepine-4-one	Down	225.0372305	3.221916667	neg	0.164096947	0.001859775
(R)-2-Hydroxy-2H-1,4-benzoxazin-3(4H)-one	Up	493.2538803	13.1769	pos	9.932378899	0.031985441
N-oleoyl phenylalanine	Up	440.3165697	14.31498333	pos	8.912549597	0.035774258
Maculosin	Up	486.2276224	10.45673333	pos	8.303702017	0.023156838
25-Hydroxytachysterol3	Up	605.3960791	14.21121667	pos	9.703066706	0.039666269
L-Methionine	Down	471.2415241	7.9601	neg	0.139673953	0.016879973
Medicanine	Up	251.2370259	12.33651667	pos	6.338042532	0.016651577
(R)-Bitalin A	Down	872.5737078	12.43791667	pos	0.104207672	0.046486462
Ivabradine	Up	348.2898338	11.43391667	pos	5.817695274	0.010387985
16-F1-PhytoP	Down	482.3598486	11.35176667	pos	0.19396787	0.003257154
Vomifoliol	Up	551.3918172	14.31498333	pos	8.082792479	0.041222645
Hippuric acid sulfate	Up	289.2162421	11.74033333	pos	6.709049728	0.028345206
PA(22:4(7Z,10Z,13Z,16Z)/PGF1alpha)	Up	530.3090199	9.455933333	neg	6.89166969	0.044746307
Isoquinoline	Up	407.2650436	7.006766667	neg	5.272723378	0.013942111
6-Succinoaminopurine	Up	448.3183575	11.02641667	pos	5.987273227	0.034535978
12-Oxo-20-hydroxy-leukotriene B4	Down	363.2529155	6.540616667	pos	0.213514085	0.003326359

Riboflavine 2',3',4',5'-tetrabutanoate	Up	525.1215194	10.65216667	neg	5.76470524	0.027504676
N-Oxalylglycine	Up	195.1379018	10.11638333	pos	6.769143661	0.045963452
9R-hydroxy-2E-decenoic acid	Up	457.2022028	11.8031	neg	4.60954165	0.009553045
N-Stearoyl Cysteine	Up	605.2384949	9.664816667	neg	4.793919861	0.015479362
Tropate	Up	492.3090954	11.19618333	neg	3.978189906	0.003333691
Methyl oxindole-3-acetate	Up	413.2327312	12.19258333	pos	5.766496419	0.037891668
12-oxo-dodecanoic acid	Up	519.4386965	13.73181667	pos	5.695202463	0.03716855
4Alpha-hydroxymethyl-5alpha-cholesta-8-en-3beta-ol	Up	265.2162592	11.82305	pos	4.55862465	0.013204371
PI(19:0/0:0)	Down	894.5817486	12.45938333	neg	0.161014054	0.049763374
Sordarin	Up	413.3781188	14.31498333	pos	4.874712077	0.018631689
4-Pyridoxic acid	Down	200.091855	2.257966667	pos	0.202730295	0.02298961
PGE3 1,15-lactone	Down	869.5149781	9.56065	neg	0.205577961	0.039953512
